# Subependymal Giant Cell Astrocytoma Without Clinical Evidence of Tuberous Sclerosis Complex: Diagnostic and Molecular Insights—Case Report

**DOI:** 10.3390/neurolint18070135

**Published:** 2026-07-14

**Authors:** José Guilherme Jasper Pickler, Hercílio Fronza Junior, Francis Rossetti Pedack, Luisa Andrade Gabardo, Gabriel Coelho Barros, Suzana Bastos Batista, Bruna Louise Silva, Paulo Henrique Condeixa de França, Rafael Roesler, Karina Munhoz de Paula Alves Coelho

**Affiliations:** 1 Department of Scientific Development and Innovation (DECIPE), Center for Anatomo-Pathological Diagnosis (CEDAP), Joinville 89201-330, Brazil; jose.pickler@univille.br (J.G.J.P.); gabriel-c-barros@hotmail.com (G.C.B.);; 2 Graduate Program in Health and Environment, University of the Joinville Region (UNIVILLE), Joinville 89219-710, Brazil; 3 Department of Teaching and Research, Hospital Municipal São José, Joinville 89202-000, Brazil; 4 Department of Mechanical Engineering, Center for Technological Sciences, Graduate Program in Materials Science and Engineering, Universidade do Estado de Santa Caratina (UDESC), Joinville 89219-710, Brazil; 5 National Science and Technology Institute for Children’s Cancer Biology and Pediatric Oncology—INCT BioOncoPed, Porto Alegre 90035-003, Brazil; 6 Department of Pharmacology, Institute for Basic Health Sciences, Federal University of Rio Grande do Sul (UFRGS), Porto Alegre 90035-003, Brazil; 7 Cancer and Neurobiology Laboratory, Experimental Research Center, Clinical Hospital (CPE-HCPA), Federal University of Rio Grande do Sul, Porto Alegre 90035-903, Brazil; 8 Center for Biotechnology, Federal University of Rio Grande do Sul, Porto Alegre 91501-970, Brazil; 9 Graduate Program in Medical Sciences, Faculty of Medicine, Federal University of Rio Grande do Sul, Porto Alegre 90035-003, Brazil

**Keywords:** subependymal giant cell astrocytoma, SEGA without TSC, tuberous sclerosis complex, intraventricular tumor, thyroid transcription factor-1, immunohistochemistry, differential diagnosis, pediatric neuro-oncology, case report

## Abstract

Introduction: A subependymal giant cell astrocytoma (SEGA) is a benign tumor typically associated with tuberous sclerosis complex (TSC), an autosomal dominant syndrome. Case report: The patient, a 15-year-old male, presented with headaches, nausea, and visual obscurations, consistent with increased intracranial pressure. Neuroimaging identified a mass in the anterior left lateral ventricle causing unilateral obstruction at the foramen of Monro. During microsurgery, smears showed a low-grade glial tumor with a biphasic mix of elongated astrocytes and large epithelioid-to-gemistocyte-like cells. Gross total resection was achieved. On permanent sections, a tumor with large polygonal, ganglioid, and gemistocytic-like cells was seen. Nuclear pleomorphism, a feature of SEGA, was present. On immunohistochemistry, the tumor was positive for glial fibrillary acidic protein (GFAP), S100, and CD34, and the cells also displayed nuclear staining for TTF-1. A diagnosis of SEGA in the absence of clinical features of TSC was established; however, definitive classification as sporadic remains limited by the lack of molecular data. Conclusions: This case highlights the importance of evaluating intraventricular masses through the integration of lineage-specific immunohistochemical panels to prevent misclassification of pleomorphic giant cells as high-grade gliomas.

## 1. Introduction

Classified as a World Health Organization (WHO) grade I circumscribed astrocytic glioma [1], SEGA, a subependymal giant cell astrocytoma, is a benign and slow-growing tumor. It usually arises in the periventricular zone [2] and tends to cluster near the foramen of Monro [3]. From an epidemiological standpoint, SEGA is more common in the pediatric population and is strongly associated with tuberous sclerosis. It occurs mainly during the first two decades of life and represents approximately 1–2% of all pediatric tumors [4]. Clinically, SEGA is widely regarded as a core feature of tuberous sclerosis complex (TSC), an autosomal dominant syndrome that drives widespread hamartoma growth [5].

Given this strong association, identifying a SEGA in a patient without germline mutations or phenotypic signs of TSC is highly unusual. When such cases arise in the absence of clinical or phenotypic evidence of TSC, they are often described as “sporadic” in the literature; however, this designation should be interpreted with caution, as somatic mosaicism in TSC genes has been proposed as a possible underlying mechanism, which may help explain the absence of other systemic manifestations in these patients [5].

In recent years, the classification of central nervous system tumors has undergone significant changes, driven by advances in molecular biology and a better understanding of the genetic alterations underlying these neoplasms. As a result, diagnostic criteria have evolved to become more comprehensive and integrated. The 2021 WHO Classification of Tumors of the Central Nervous System (CNS5) currently recommends the combination of traditional histology with molecular and immunohistochemical profiling, with thyroid transcription factor-1 (TTF-1) proving particularly valuable in the differential diagnosis of intraventricular tumors [1,6,7,8].

## 2. Case Presentation

A 15-year-old male presented with signs of increased intracranial pressure, complaining of refractory headaches, nausea, and transient visual obscurations. Neuroimaging identified a circumscribed, contrast-enhancing mass in the anterior left lateral ventricle causing unilateral obstruction of the foramen of Monro.

**Figure 1:**
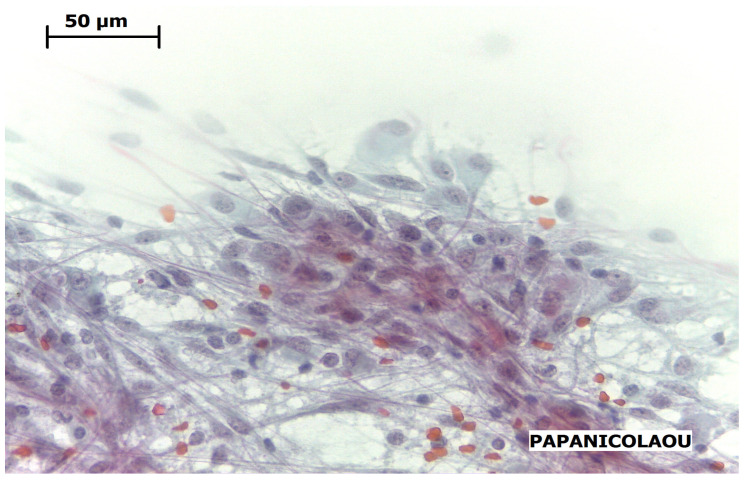
Intraoperative cytology smear stained with Papanicolaou showing a low-grade glial tumor with a biphasic pattern composed of elongated astrocytic cells admixed with larger epithelioid to gemistocytic-like cells in a delicate fibrillary background (Papanicolaou, ×40).

Microsurgical resection was performed to treat the obstructive hydrocephalus. Intraoperative smears showed a low-grade glial tumor featuring a biphasic mix of elongated astrocytes and large epithelioid to gemistocytic-like cells within a delicate fibrillar matrix (Figure 1). Based on these intraoperative findings, gross total resection was successfully achieved.

**Figure 2:**
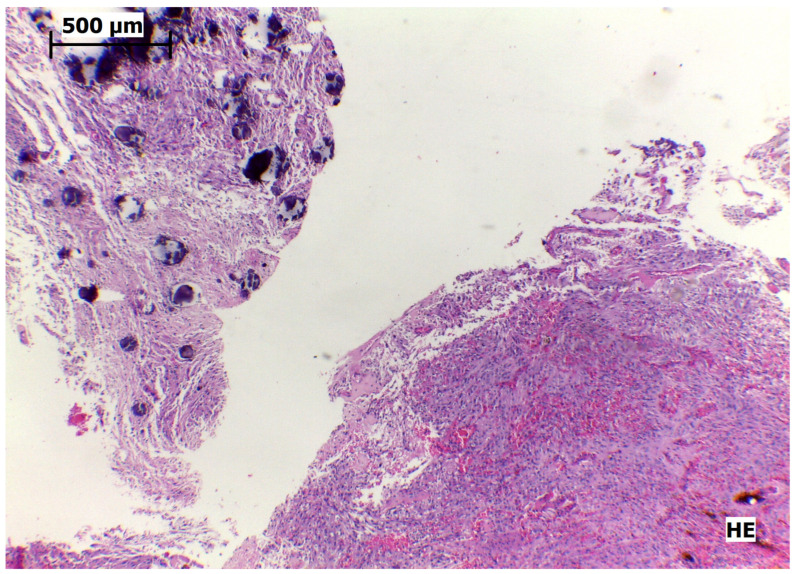
Histologic sections show a tumor composed of mixed cell populations, including large polygonal and spindle cells, arranged in fascicular and nesting patterns within a delicate fibrovascular stroma. Focal stromal calcifications are present (H&E, ×10).

Histologic sections demonstrated a circumscribed tumor with a heterogeneous population of large polygonal, ganglioid, and gemistocytic-like cells alongside spindle-shaped glial cells. The cells formed complex fascicular and nesting patterns separated by fine fibrovascular septa. Nuclear pleomorphism typical of SEGA was present, including prominent nucleoli and occasional multinucleation, though mitotic activity remained low. Focal stromal calcifications were noted, but there was no microvascular proliferation or coagulative necrosis (Figure 2).

**Figure 3:**
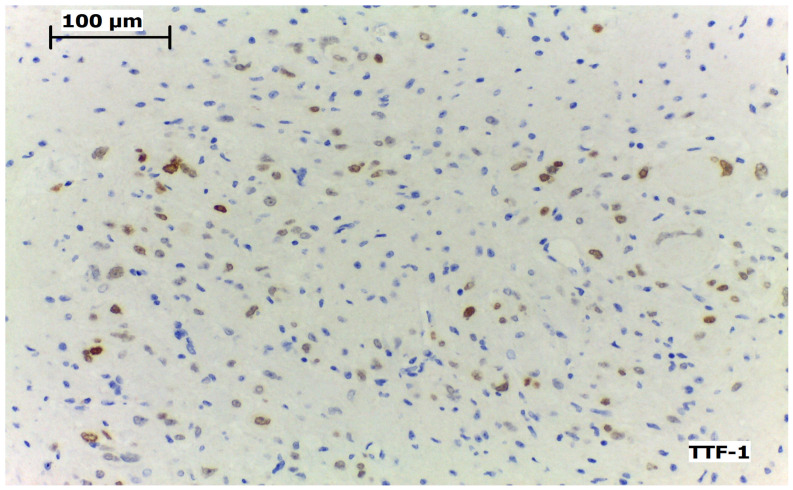
Immunohistochemical staining shows strong, diffuse nuclear expression of TTF-1 in tumor cells (TTF-1, ×20).

On immunohistochemistry, the tumor was widely positive for glial fibrillary acidic protein (GFAP), S100, and CD34. The neoplastic cells also displayed strong, diffuse nuclear staining for TTF-1 (Figure 3). Epithelial membrane antigen (EMA) and CD99 staining were weak and focal. A Ki-67 index of 2–3% was consistent with an indolent clinical course.

After confirmation of the SEGA diagnosis, the patient underwent a targeted systemic workup to evaluate underlying syndromic associations. He lacked any classic clinical stigmata of tuberous sclerosis complex (TSC). Additionally, his family history was negative for neurocutaneous syndromes. These findings supported a diagnosis of SEGA in the absence of clinical evidence of TSC. However, definitive classification as a sporadic case remains limited by the lack of molecular data.

## 3. Discussion

Our patient presented with a SEGA without clinical evidence of TSC, a rare neuro-oncological presentation with very few pathologically and genetically confirmed cases reported in the literature [2,9,10]. The age of presentation is similar to that described in the literature, occurring in the first and second decades of life, except for the description of a 33-year-old patient by Stavrinou et al. Regarding symptomatology, the reports evaluated are similar to this case, with patients presenting symptoms of increased intracranial pressure. Table 1 contextualizes our case in relation to recently reported cases regarding their symptoms, histological findings, and clinical outcomes [9].

Beaumont et al. report a case with a patient of similar age and symptomatology to ours and consider SEGA, central neurocytoma, choroid plexus tumor, astrocytoma, and meningioma as possible radiographic differential diagnosis. The hypothesis of tuberous sclerosis was also ruled out due to the absence of skin signs such as hypomelanotic macules, shagreen patches, or facial angiofibromas [2].

While morphologically identical to syndromic TSC-associated tumors, the underlying genomic architecture of these cases may differ. In classical TSC, germline mutations in either TSC1 or TSC2 disrupt the hamartin-tuberin complex. This leads to constitutive mTOR hyperactivation, driving cell proliferation, and cytomegaly characteristics of the tumor [5]. In contrast, SEGA’s arising in patients without clinical evidence of TSC have been hypothesized to result from localized somatic mosaicism or biallelic somatic mutations restricted to neural progenitor populations, although this cannot be confirmed in the absence of molecular testing [5]. Complete surgical resection is still the primary goal. However, identifying the underlying mTOR dysregulation has practical clinical value, as mTOR inhibitors are an effective targeted therapy for unresectable or residual disease [5].

The differential diagnosis of intraventricular tumors in this context includes central neurocytoma, ependymoma, high-grade gliomas, and choroid plexus tumors. Central neurocytomas typically show uniform round cells with neuronal differentiation and synaptophysin positivity, lacking the characteristic admixture of ganglioid and gemistocytic-like cells seen in SEGA. Ependymomas may arise in a similar location but generally exhibit perivascular pseudorosettes and a distinct immunophenotype. High-grade gliomas may show marked pleomorphism; however, they are usually associated with high mitotic activity, microvascular proliferation, and necrosis, which were absent in this case.

Histologically, SEGAs present a mixed morphological spectrum, including spindled glia, gemistocytic-like cells, and ganglioid cells, which can complicate the diagnosis [1]. Relying solely on cytological pleomorphism often leads to confusion with central neurocytomas or higher-grade gliomas. In the case presented, histology revealed mixed cell populations, including large polygonal and fusiform cells, and cytology showed elongated astrocytic cells mixed with larger cells, similar to epithelioid and gemistocytic cells. These findings are shown in Figure 1 and Figure 2. In this context, the integration of morphology and immunohistochemistry is essential for accurate diagnosis. For our patient, strong and diffuse nuclear positivity for TTF-1 was key to securing the diagnosis [7,8], as shown in Figure 3.

This TTF-1 expression also provides insight into the tumor lineage. Although typically associated with thyroid and pulmonary development, TTF-1 appears transiently in the embryonic ventral forebrain [8]. Studies of human fetal neuroanatomy localize it to the medial ganglionic eminence (MGE), a known source of GABAergic interneuron progenitors [7]. The immunophenotypic overlap between the MGE and SEGA suggests that the tumor arises from a persistent, localized progenitor population within the MGE [7,8]. This developmental link explains why SEGAs almost exclusively develop in the periventricular zone along the caudothalamic groove [8]. This spatial correlation between MGE-derived progenitors and ventricular zone tumors supports, but does not confirm, a potential developmental origin hypothesis for SEGA. However, this hypothesis remains speculative and should be interpreted with caution, particularly in the absence of molecular validation.

It is important to emphasize that this is a single case report, with limitations inherent to the method, and it is not possible to generalize the findings. A key limitation of this study is the lack of postoperative follow-up data, as the patient did not continue clinical evaluation at our institution, precluding assessment of long-term outcomes and recurrence. The limitations of this study also include the absence of molecular profiling to confirm TSC. Therefore, definitive classification as a truly sporadic case remains limited. The literature describes the uncertainty regarding whether similar cases involve a spontaneous solitary tumor or another form related to TSC [12].

## 4. Conclusions

The overall clinical prognosis for SEGAs occurring in the absence of clinical evidence of TSC is favorable following successful gross total resection [3], mirroring the comparatively benign natural history of syndromic cases. However, given the inherently complex and incompletely characterized somatic mutational landscape driving these tumor, long-term postoperative magnetic resonance imaging (MRI) surveillance remains warranted to monitor for delayed recurrence [2,9,10]. In selected cases, complementary diagnostic approaches, including cerebrospinal fluid (CSF) cytology, may provide additional information for disease assessment [14]. This case underscores the diagnostic importance of integrating modern lineage-specific immunohistochemical panels, particularly TTF-1, in the histopathological evaluation of complex intraventricular masses. The accurate identification of such lineage-restricted markers helps to prevent clinical misclassification of highly pleomorphic giant cells as high-grade gliomas [7,8]. It also provides insights into the fundamental ontogeny of central nervous system tumors, although such interpretations should be made cautiously in the absence of molecular validation.

## Figures and Tables

**Table 1:** Clinical Characteristics and Outcomes of Representative Sporadic SEGA Cases (Adapted from [9] Holler et al., 2025).

Citation	Age/Sex	Presenting Symptoms	Histological Findings	Clinical Outcome
[11]	20/F	Medically refractory headache	Large gemistocytic cells with abundant cytoplasm and fibrillated spindle cells. Positive for glial fibrillary acidic protein (GFAP), while large cells lacked detectable GFAP. Staining for S100 protein, neuron-specific enolase, neurofilament (NFL), and synaptophysin (SYN) were negative.	Complete resolution post resection; no recurrence at 28 months
[12]	33/M	Headache, papilledema, nuchal rigidity	Neoplastic cells with astroglial differentiation or large cells with rounded nuclei intermingled with spindle cells and multinucleated giant cells. An abundance of mast cells and histiocytes was observed. There was limited focal reaction for GFAP staining, strong positivity for S100 protein, and weak reactivity for vimentin, synaptophysin, and chromogranin.	Complete resolution post resection
[13]	25/F	Headache	Cells with variable morphology. In some areas, the cells appeared spindle-shaped with long processes running in fascicles. The tumor was composed of pleomorphic cells with multinucleation and abundant eosinophilic cytoplasm. Rare mitotic figures were seen. Extremely high vascularity was noted A small fragment of the tissue revealed calcifications. GFAP was positive and synaptophysin revealed scattered positive cells.	Resolution post resection; no recurrence at 9 months
[3]	22/F	Visual disturbance, severe tinnitus	Nonuniform staining for GFAP and positive neurofilament and synaptophysin staining. Staining for neuronal nuclear protein (Neu-N) was not prominent. Staining for epithelial membrane antigen (EMA), isocitrate dehydrogenase 1 (IDH-1) (R132H mutation), BRAF (V600E mutation), and p53 were also negative.	Near-total resection; required subsequent ventriculoperitoneal shunt
Current Case	15/M	Headache, emesis, visual obscurations	Heterogeneous population of large polygonal, ganglioid, and gemistocytic-like cells. The cells formed complex fascicular and nesting patterns. Presence of nuclear pleomorphism.	Gross total resection; resolution of hydrocephalus

## Data Availability

The data supporting the findings of this study are available from the corresponding author upon request.

## Abbreviations

The following abbreviations are used in this manuscript:

SEGA	Subependymal giant cell astrocytoma
TSC	Tuberous sclerosis complex
TTF-1	Thyroid transcription factor-1
GFAP	Glial fibrillary acidic protein
WHO	World Health Organization
EMA	Epithelial membrane antigen
H&E	Hematoxylin and Eosin
